# Sixty years of conjecture over a urinary biomarker: a step closer to understanding the proposed link between anxiety and urinary pyrroles

**DOI:** 10.1093/labmed/lmad086

**Published:** 2023-09-12

**Authors:** Angela Sherwin, Ian C Shaw

**Affiliations:** School of Physical & Chemical Sciences; School of Psychology, Speech & Hearing, University of Canterbury, Christchurch, New Zealand; School of Physical & Chemical Sciences

**Keywords:** Mauve Factor, kryptopyrrole, hydroxypyrrole, biomarker for anxiety, urinary biomarker, generalized anxiety disorder

## Abstract

**Objective:**

For over 60 years there has been conjecture about the identity of an Ehrlich’s test positive pyrrole (Mauve Factor) reputed to be a biomarker for psychological disorders, including anxiety. We reviewed studies that attempt to identify Mauve Factor and subjected authentic standards of the 2 main candidates, kryptopyrrole and hydroxypyrrole, to the Ehrlich’s reaction.

**Methods:**

Modified Ehrlich’s test for kryptopyrrole and hydroxypyrrole were applied to urine samples from 10 volunteers, anxious and nonanxious.

**Results:**

Based on the mechanistic chemistry of Ehrlich’s reaction and reactions of the 2 compounds, Mauve Factor cannot be hydroxypyrrole. Analyses of urine samples from volunteers, identified by the Generalized Anxiety Disorder - 7 item scale (GAD-7 ≥10; n = 5) and control urine samples (GAD-7 <10; n = 5) using a kryptopyrrole calibration graph, show that concentrations are similar in both groups.

**Conclusion:**

Kryptopyrrole may be the elusive Mauve Factor. Its possible origin from stercobilin via gut microbiome–mediated metabolism, its link to gut-mediated neurological effects via γ-aminobutyric acid (GABA) receptors, and its predicted interaction with Zn^2+^ and consequent impact on zinc homeostasis are discussed. The GAD-7 scale does not differentiate between state and trait anxiety and as such, the minimal difference in pyrrole levels between volunteer groups requires further study.

## Introduction

Laboratory tests for biomarkers may be required more often in the future as personalized medicine becomes more widely used. Psychological disorders (eg, anxiety) are common, and therefore an easily applied laboratory test to aid in determining treatment options would be a useful adjunct to the available diagnostic tools.

Anxiety disorders are common worldwide, with a prevalence of 2.4% to 29.8% depending on age, gender, culture, and personal and national issues such as conflict and economic status.^1^ Since there are likely to be biochemical aberrations underpinning this spectrum of psychological disorders, it is feasible that biochemical markers might be useful for screening and diagnosis of anxiety.

**Figure 1.**
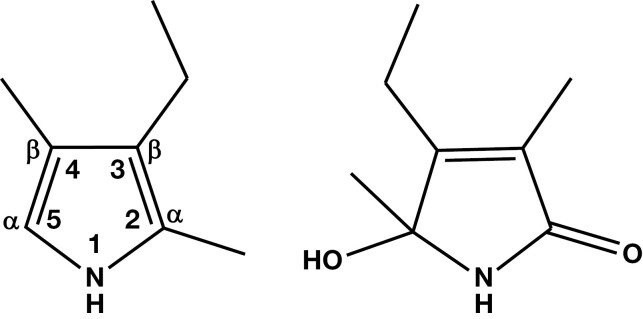
Proposed molecular structures of Mauve Factor. Left: 2,4-dimethyl-3-ethylpyrrole (kryptopyrrole; Irvine et al^3^). Right: 2,4-dimethyl-3-ethyl-2-hydroxy-2,5-dihydropyrrol-2-one (hydroxypyrrole [HPL]; Irvine^5^). The pyrrole ring numbering system and α- and β-carbons are also shown.

Approximately 60 years ago, D.J. Irvine showed that urine from patients with psychological disorders gave a characteristic mauve reaction with Ehrlich’s reagent^2^; this was termed Mauve Factor. Irvine’s group later identified the urinary compound as 2,4-dimethyl-3-ethylpyrrole (kryptopyrrole; FIGURE 1), following its chromatographic isolation from urine and mass spectrometry.^3^ Sohler et al^4^ confirmed Irvine’s identification of Mauve Factor and showed that it has a sedative effect on the central nervous system of rabbits.

Further studies by Irvine^5^ reidentified Mauve Factor as 2,4-dimethyl-3-ethyl-2,4-dihydropyrrol-4-one (hydroxypyrrole [HPL]; FIGURE 1).

The change in identity of the elusive Mauve Factor perhaps reflects the analytical methodology available at the time, and very importantly, the instability of the group of pyrroles to which Mauve Factor appeared to belong.

Hydroxypyrrole has been reported (although variously named due to changes in chemical nomenclature) in blood and urine^6-9^ and in cerebrospinal fluid^6^ but has not been reliably associated with a specific clinical disorder. However, its association with anxiety has been proposed^10^; such an association is attractive because it might be a useful biomarker in determining treatment options for anxiety.

This article was designed to review the historical literature in the area, highlight the uncertainties of the chemical structure of Mauve Factor, and apply the methodology as a proof of concept for the value of studying urinary pyrroles in relation to anxiety.

### Urinary Pyrrole Nomenclature

The chemistry of pyrroles in the urine of patients with anxiety has spanned some 60 years. During this time, there have been significant changes in chemical nomenclature and advances in experimental methodologies. Initially, scientists referred to the compounds as “Mauve Factor,” or similar because of their color reactions with Ehrlich’s reagent, later naming them specifically according to knowledge about their molecular identities at the time. Not only have the names of the compounds changed as a better understanding of their structures has developed, but also the standard chemical nomenclature has changed during this time. This can lead to significant confusion; therefore, in this study, we have used the generic term “urinary pyrroles” throughout unless we are referring to a specific compound, in which case we use the pyrrole ring numbering system shown in FIGURE 1.

### Biochemical Origins of HPL

**Figure 2.**
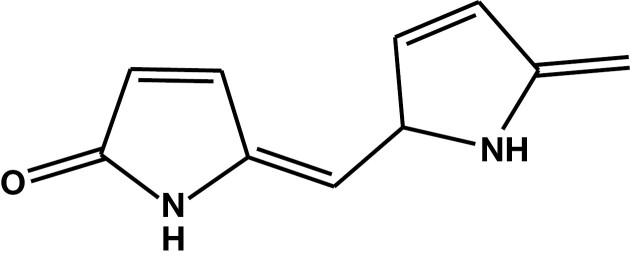
A dipyrrolic fragment of bilirubin from the catabolism of heme proposed by Brown and King.^11^

There is significant conjecture about the biochemical origins of HPL. However, it is tempting to associate it with the biodegradation of heme because of the similarities in molecular structure between HPL and heme biodegradation products (FIGURE 2). At about the same time that Irvine postulated the existence of HPL,^5^ Brown and King^11^ proposed the mechanism of heme catabolism via biliverdin to bilirubin followed by bilirubin’s further breakdown to a series of dipyrrolic fragments (FIGURE 2), which might conceivably be metabolic precursors of HPL.

### Pyrroles in Psychological Disorders

An interest in concentrations of Mauve Factor in psychological disorders led O’Reilly et al^12^ to report that of 200 patients admitted to a psychiatric ward, 89 (44.5%) were urinary Mauve Factor–positive. In addition, O’Reilly et al^13^ showed that approximately 11% of the general population and 41.8% of the psychiatric population were Mauve Factor–positive. Furthermore, “neurotic” schizophrenia patients who were Mauve Factor–positive following treatment with nicotinamide (vitamin B3) became Mauve Factor–negative, which suggests that Mauve Factor can be used as an indicator of treatment success.^14^ Pfeiffer and Iliev^15^ used similar methodology to monitor treatment and found that pyridoxal-5’-phosphate (vitamin B6) was also an effective treatment. Other studies have shown a correlation between psychological disorders and urinary pyrroles, including anxiety,^10^ depression,^10,16^ attention deficit hyperactivity disorder/attention deficit disorder,^17^ autism,^17^ and bipolar disorder.^16^ Although Gorchein^8^ found no difference between schizophrenia and general medical patients, this might reflect the anxiety levels of the hospitalized patients tested.

As a result of these and other studies that show links between an Ehrlich’s positive compound in urine (termed Mauve Factor or identified as 1 of 2 pyrrole derivatives, namely HPL or kryptopyrrole [FIGURE 1]) and various psychological disorders (including anxiety), a plethora of commercial services (eg, Riordan Clinic,^18^ Nutripath,^19^ DHA Laboratory,^20^ Amanda Nutrition,^21^ Perpetual Wellbeing,^22^ SAFE Analytical Laboratories,^23^ and Applied Analytical Laboratories^24^) have been established to carry out the Ehrlich’s test (or other unspecified analyses) on urine samples of individuals suspected of suffering from a psychological disorder, including anxiety, as a means of indicating treatment (often dietary supplements or herbal remedies). These services variously claim to be analyzing for HPL, kryptopyrrole, pyrroles generally, or give no clear indication of what they are testing for. Similarly, of the multitude of scientific publications purporting to study various pyrroles in urine of patients with psychological disorders, most have ill-defined descriptions of the analytical methodology, in particular, its validation,^25-30^ and do not appear to use authentic standards (eg, HPL) for their analytical validation; as a result, their findings are unreliable. At the time of writing, the web-based companies that offer analyses to determine therapy rarely, if ever, describe their analytical methodology.

### Chemistry of Ehrlich’s Test

**Figure 3.**
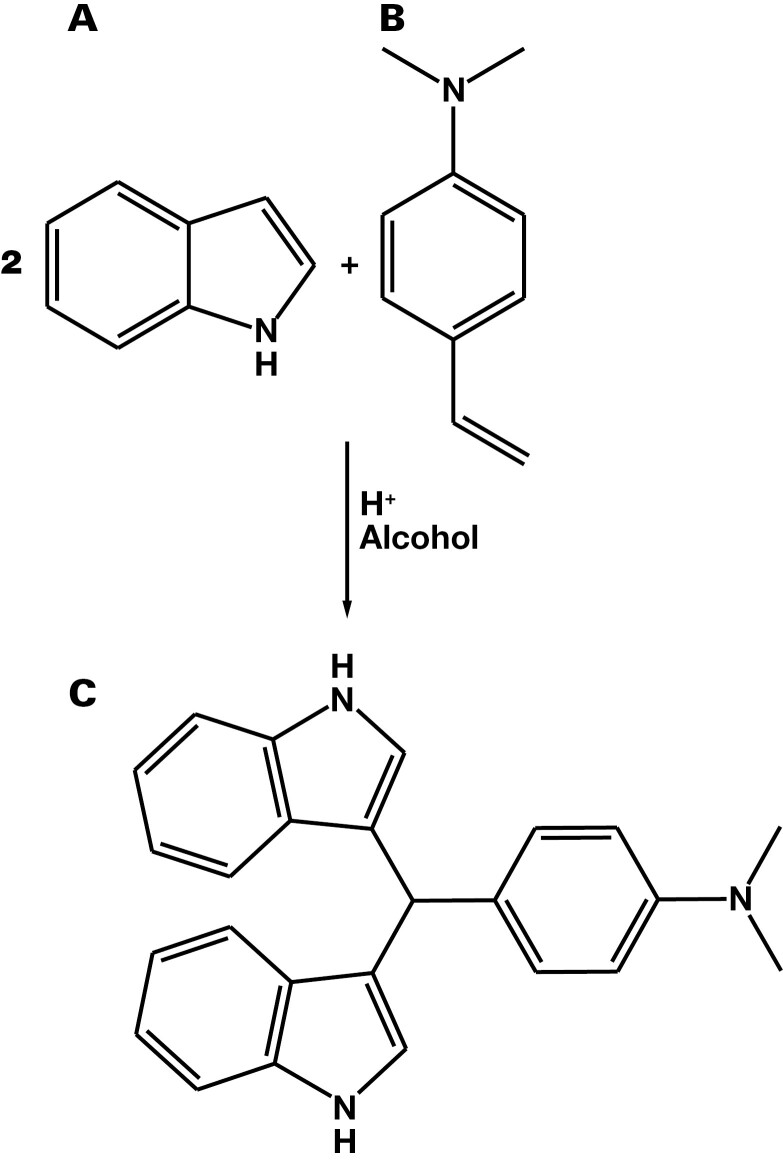
Ehrlich’s reaction: the β-carbon of indole (A) reacts with 4-dimethylaminobenzaldehyde (DMAB) (B) to form purple β-bis(indolyl)dimethylaminobenzyl methane (C).^32^

Based on Ehrlich’s earlier work, Morton^31^ noted that all pyrroles with unsubstituted α-carbons react with 4-dimethylaminobenzaldehyde (DMAB) to produce a characteristically colored compound. They also noted that the “reaction is partially successful with compounds that have a free beta position” and that α-carboxy or α-ester-substituted pyrroles gave a reaction when heated^31^ (p 68). Recently Lamb et al^32^ unraveled the mechanism of the reaction between DMAB and indole (FIGURE 3) and showed that reaction position is dependent on the chemical environment but that the conventional Ehrlich’s reagent (ie, DMAB in acid solution) results in a reaction at the β-carbon of the pyrrole ring (FIGURE 1). This means that in Morton’s terms,^31^ either a free α- or β-carbon is required, and in the terms of Lamb et al,^32^ a free β-carbon is required for the Ehrlich’s reagent to react with a pyrrole. Hydroxypyrrole has both α- and β-carbons fully substituted, and its carbon-carbon double bond is in the wrong position (ie, it is a 2,4-dihydropyrrole), whereas kryptopyrrole has an unsubstituted α-carbon. Therefore, HPL is unlikely to give a positive Ehrlich’s reaction, whereas kryptopyrrole is likely to react.

To determine pyrroles in urine, a modified Ehrlich’s test is used.^33^ In short, Ehrlich’s reagent is prepared by dissolving DMAB in methanol and acidifying the methanolic solution with sulfuric acid. Pyrroles are extracted from urine samples with trichloromethane, thus leaving other Ehrlich’s positive compounds (eg, urobilinogen) in the aqueous phase. The trichloromethane extract is mixed with Ehrlich’s reagent and after a brief incubation period, the absorbance is read at 540 nm (see Materials and Methods).

As outlined above, there is significant conjecture about the presence and identity of pyrroles in urine and their link to psychological disorders (including anxiety). Two key questions remain unanswered: what does the modified (including trichloromethane extraction) Ehrlich’s test measure in urine? And does the test reliably predict an intervention of therapeutic value?

In this study, we investigated the reaction of HPL and kryptopyrrole with Ehrlich’s reagent and applied the modified Ehrlich’s test to urine samples obtained from volunteers who were not suspected of suffering from a psychological disorder (including anxiety) using an authentic kryptopyrrole calibration graph to determine urinary kryptopyrrole concentrations. We discuss our findings in the context of psychological disorder (including anxiety) management.

## Materials and Methods

### Chemicals

All chemicals were general laboratory reagent grade and purchased from Merck except HPL (95% purity; Chem-Space.com), kryptopyrrole (97% minimum purity; LabSupply.co.nz), and trichloromethane (≥99.85% purity; ThermoFisher Scientific). Both HPL and kryptopyrrole were supplied with proton nuclear magnetic resonance or mass spectroscopic data, which confirmed their molecular structures.

### Modified Ehrlich’s Reagent

Modified Ehrlich’s regent was prepared as described by Sohler et al.^33^ In brief, DMAB (1 g) was dissolved in ice cold methanol (80 mL), concentrated sulfuric acid (10 mL) was added drop-wise, and the solution made up to 100 mL with methanol. The reagent was stored at 4°C and used within 24 hours.

### HPL and Kryptopyrrole Calibration Graphs

Hydroxypyrrole (5 mL, 1-70 μg/mL [aq]) was extracted with trichloromethane (5 mL) in glass stoppered test tubes (25 mL). The trichloromethane layer was pipetted into a test tube, dried with anhydrous sodium sulfate (0.5 g), and modified Ehrlich’s reagent (1 mL) was added (to 4 ml extract). The mixture incubated at room temperature for 30 minutes, then its absorbance at 540 nm was determined (Model T75+ UV/visible spectrophotometer, Bio-Strategy).

Kryptopyrrole (0.5 mg/mL in ethanol) was pipetted into a series of tubes and made up to 210 μL with ethanol and distilled water (4.79 mL) was added to give a range of final kryptopyrrole concentrations (1-5 μg/mL). Trichloromethane (5 mL) was added and the procedure described above for HPL was followed.

All HPL and kryptopyrrole concentrations were prepared and analyzed in triplicate. Results are expressed as mean ± SD.

### Ehrlich’s Spot Test for HPL

Hydroxypyrrole (1 mg) was dissolved in trichloromethane (1 mL), Ehrlich’s reagent (250 µL) was added, the mixture was incubated at room temperature for 30 minutes, and its absorbance at 540 nm determined.

### Volunteer Inclusion Criteria

Volunteers were screened using the Generalized Anxiety Disorder - 7 item scale (GAD-7),^34^ a 7-point Likert scale with a maximum score of 21 (10 or above was the cut off point for anxiety for this study), and a general health questionnaire. All volunteers reported that they had no general health issues and were not taking any medications. Only females were chosen to help avoid confounding variables, as there is some suggestion that current psychometrics do not adequately pick up anxiety in men.^35,36^

### Urine Samples

Midstream urine samples (~50 mL) were collected from female volunteers (n = 5, age 34 ± 11.3 years [mean ± SD]) with no known psychiatric disorders and GAD-7 <10 and females with symptoms of anxiety (n = 5, age 42.8 ± 8.2 years) and GAD-7 ≥10 (University of Canterbury Human Ethics Committee approval 17/STH/241). The urine samples were preserved with ascorbic acid (final concentration in urine ~0.1 g/mL) to inhibit pyrrole oxidation. Samples were stored at −18°C prior to analysis.

### Urine Analysis

Urine samples (5 mL) were extracted with trichloromethane (5 mL). The trichloromethane extracts were analyzed with modified Ehrlich’s reagent as described for HPL and kryptopyrrole calibration graphs above. All samples were analyzed in triplicate.

## Results and Discussion

### Reaction of HPL and Kryptopyrrole with Modified Ehrlich’s Reagent

**Figure 4.**
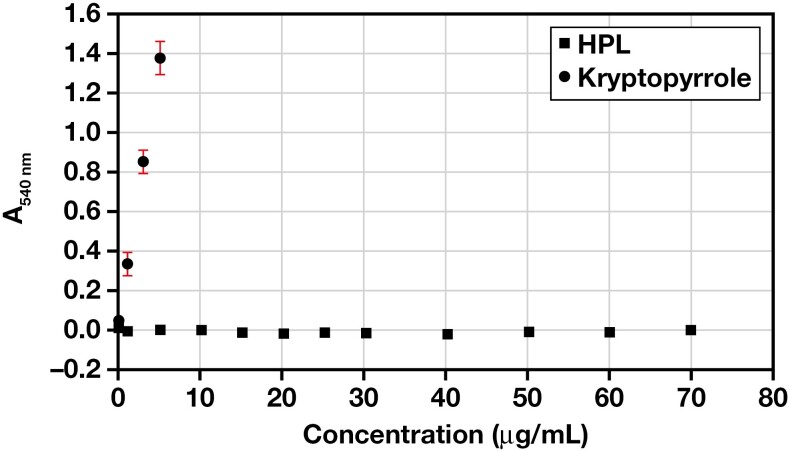
Reaction of hydroxypyrrole (HPL) and kryptopyrrole with modified Ehrlich’s reagent. This clearly shows that HPL does not react with 4-dimethylaminobenzaldehyde (DMAB) and that kryptopyrrole reacts in a concentration-related manner. Results are shown as mean ± SD (n = 3).

Kryptopyrrole reacted with modified Ehrlich’s reagent in a concentration-dependent manner that obeys Beer’s Law between 1 and 5 μg/mL (FIGURE 4). On the other hand, HPL did not react with modified Ehrlich’s reagent (FIGURE 4) at any of the concentrations (1 to 70 μg/mL) tested. This lack of reaction with modified Ehrlich’s reagent was confirmed with a higher concentration (1 mg/mL) of HPL, which also gave no color reaction (A_540 nm_ = 0.035). This is not surprising, as HPL does not have a free α- (or β-) carbon that is required for reaction with DMAB.^31,32^ Kryptopyrrole does have a free α-carbon and therefore would be expected to react with DMAB.

This finding contradicts previous studies^5,25,27,28,37–42^ that identify the urine component responsible for the Ehrlich’s mauve reaction as HPL. Interestingly, none of the previous studies used a pure, authentic HPL standard and so based their conclusions on a possible misinterpretation of the chemistry of the Ehrlich’s reaction. It is now clear that urinary Mauve Factor is not HPL but could be kryptopyrrole.

Rainforest et al^29^ confirmed the importance of kryptopyrroles [sic] as a biomarker for psychological disorders in Australian clinical practice. However, they did not specify what was measured in the urine samples they reported on. Therefore, even the most current publication on urinary pyrroles in psychological disorders at this time purports to be measuring kryptopyrroles but might not be. The confusion continues.

### Importance of the Identity of the Urinary Pyrrole(s) in Anxiety

The use of a positive urine reaction with Ehrlich’s reagent as an indicator of anxiety would be a useful tool for diagnosis. Additionally, identification of the specific urinary pyrrole(s) might help our understanding of the biochemistry of anxiety. For example, reduced serum Zn^2+^ concentrations have been shown in cases of anxiety, and zinc therapy has resulted in reduction in the symptoms of anxiety.^43^ The importance of zinc (likely as Zn^2+^) in anxiety might be related to its role in γ-aminobutyric acid (GABA)ergic neurotransmission^44,45^ via glutamate regulation.^43^ If a specific pyrrole has a role in Zn^2+^ homeostasis, it would be a key biochemical marker and might indicate a biochemical mechanism underpinning anxiety.

**Figure 5.**
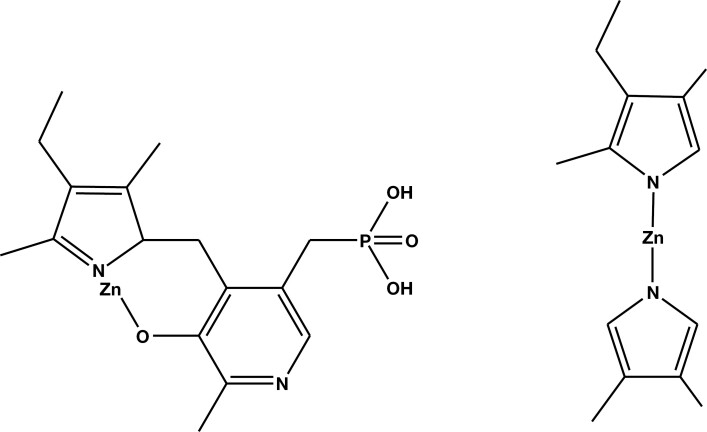
Postulated structure of the pyridoxal phosphate/kryptopyrrole-zinc chelate^46^ (left) and possible, but unlikely, zinc-kryptopyrrole complex (right).

It has been suggested that zinc (likely as Zn^2+^) is excreted as a pyridoxal phosphate kryptopyrrole chelate^46^ (FIGURE 5). This might be a mechanism of Zn^2+^ depletion, resulting in reduced serum Zn^2+^ concentration, with its concomitant implications for GABA-mediated neurotransmission and perhaps its role in anxiety. The fact that a co-chelate between Zn^2+^ and pyridoxal phosphate/kryptopyrrole is the Zn^2+^ excretory vector would mean that kryptopyrrole is a biomarker for anxiety. The question is, would the postulated zinc pyridoxal phosphate/kryptopyrrole complex react with Ehrlich’s reagent to give the characteristic mauve color? This is unlikely because the pyrrole ring does not have a free α- (or β-) carbon. On the other hand, would excess urinary kryptopyrrole (ie, that remaining after reaction with pyridoxal phosphate) indicate loss of Zn^2+^ via the chelate pathway? If this were the case, the presence of kryptopyrrole in urine would likely indicate loss of zinc via the urinary route.

It is possible but unlikely that a zinc-kryptopyrrole complex (FIGURE 5) might be formed and that this underpins the mechanism of Zn^2+^ loss via urine. This is considered unlikely because the pyrrole nitrogen in kryptopyrrole is a very weak base (pyrrole p*K*_b_ = 13.6) and so does not support its interaction with Zn^2+^.

### Analysis of Urine Samples

Modified Ehrlich’s analysis of urine samples from volunteers showed the presence of an Ehrlich’s-positive compound (or compounds) that absorbs at 540 nm (Table 1). If our hypothesis that this compound is kryptopyrrole is correct, using the kryptopyrrole calibration graph (FIGURE 4), it corresponds to a urine kryptopyrrole concentration of 0.40 ± 0.065 μg/mL (mean ± SD) for the nonanxious volunteers and 0.42 ± 0.083 for the anxious volunteers. Both are considered high.^17,20,42,47^ On the face of it, the difference between the 2 groups in this study is interesting and surprising. Using an independent samples *t*-test with all assumptions met to determine whether there were differences in pyrrole absorbance between the 2 groups we see that there is no statistically significant difference, M = 0.16, 95% CI [0.09, 0.12], t(8) = 0.341, *P* = .7. It is important to note that our study was designed to investigate the application of the methodology to urine samples to inform future study rather than differentiate between the 2 groups. In addition, the GAD-7 scale does not differentiate between state and trait anxiety. It is possible that the nonanxious volunteers were in a state of anxiety not picked up by the GAD-7.

**TABLE 1.** Modified Ehrlich’s analysis of urine samples from female volunteers with GAD-7^a^

Volunteer	A_540 nm_ Mean ± SD	Kryptopyrrole equivalents µg/mL
GAD-7 <10		
1	0.075 ± 0.009	0.36
2	0.066 ± 0.006	0.31
3	0.098 ± 0.008	0.47
4	0.092 ± 0.010	0.44
5	0.094 ± 0.011	0.45
Mean ± SD		0.41 ± 0.065
GAD-7 ≥10		
1	0.092 ± 0.014	0.44
2	0.104^b^	0.49
3	0.081 ± 0.004	0.39
4	0.062 ± 0.011	0.30
5	0.103 ± 0.013	0.49
Mean ± SD		0.42 ± 0.083

GAD-7, Generalized Anxiety Disorder - 7 item scale.

^a^GAD-7 <10 (n = 5; mean ± SD age = 32.5 ± 10.8 years) and GAD-7 ≥10 (n = 5; mean ± SD age = 42.8 ± 8.2 years) showing kryptopyrrole equivalents.

^b^Single sample.

A cross-sectional analysis of pyrroles in psychiatric disorders^28^ suggested that there is a correlation between histamine levels and pyrrole levels. Histamine itself is unlikely to react with Ehrlich’s reagent as it does not have a free β-carbon.^31^ Clearly, more research is needed to confirm the identity of the Ehrlich’s-positive compound and understand its potential value as a clinical biomarker. This is made difficult by the likely instability (stability data not presented) of kryptopyrrole when extracted from urine.

**Figure 6.**
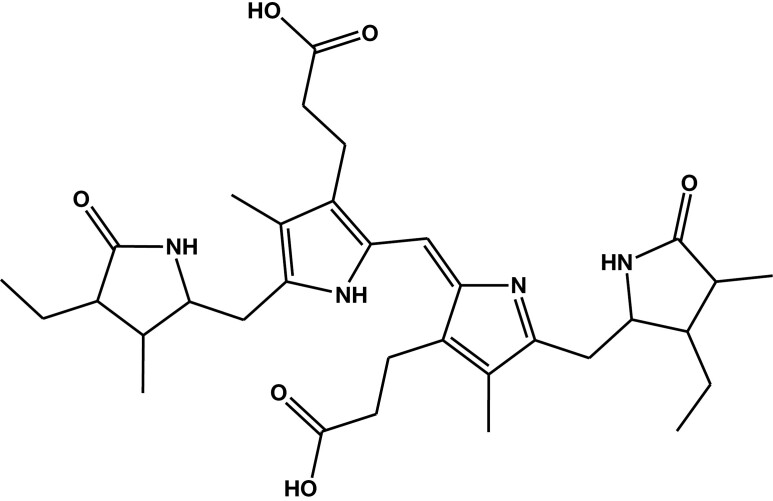
Stercobilin: the two 3-ethyl-4-methyl-3,5-dihydropyrrol-2-one moieties at each end of the molecule have significant structural similarities to hydroxypyrrole (HPL) and kryptopyrrole (see FIGURE 1).

It has been suggested that stress-related gut permeability changes might account for stress-associated HPL in blood.^9^ This too is feasible, as bilirubin is excreted as a glucuronide conjugate in bile and gut microbiome β-glucuronidase releases the aglycone, which is normally further metabolized by the gut microbiome to stercobilin and eliminated in feces.^48^ Stercobilin (FIGURE 6) is likely to be in part further degraded by the gut microbiome. Interestingly, stercobilin has parts of its molecular makeup that have remarkable structural analogy to HPL and kryptopyrrole (compare FIGURES 1 and 6).

Furthermore, recent research has shown that stercobilin is absorbed from the gut in mice and that its presence in blood is linked to proinflammatory activity.^49^ Interestingly, peripheral proinflammatory cytokines (eg, interleukins) have been linked to generalized anxiety disorder in Chinese patients^50^; this might furnish a link between leaky gut, stercobilin absorption, inflammatory response, and generalized anxiety disorder, which in turn presents a possible molecular association with alkylated pyrroles (perhaps derived from stercobilin).

If the Ehrlich’s-positive compound in urine is kryptopyrrole, this might link its association with anxiety to a gut-mediated mechanism involving the biodegradation of stercobilin by the gut microbiome leading to a stercobilin-mediated inflammatory response.

## Conclusion

Our study is the first to use authentic standards of HPL and kryptopyrrole and shows that urinary Mauve Factor is not HPL. Its identity remains unproved, but the molecular requirements for reaction with DMAB are met by kryptopyrrole (ie, α, β-unsubstituted pyrrole) and so kryptopyrrole is a likely candidate for the elusive Mauve Factor.

It is interesting to speculate that kryptopyrrole has its biochemical origins in stercobilin and might indicate a link with leaky gut syndrome, which has been reported to be associated with anxiety.^50^ If kryptopyrrole is excreted in urine in clinical anxiety, the postulated co-chelate with pyridoxal phosphate and zinc^46^ would suggest a biochemical mechanism of anxiety, point to a dietary supplement-based treatment, and present a biomarker for diagnosis; however, because there is no pyrrole concentration difference between control and anxious urine samples in this small sample set, more work is needed to assess the usefulness of kryptopyrrole as an anxiety biomarker.

## Abbreviations:

GAD-7: Generalized Anxiety Disorder - 7 item scale
HPL: hydroxypyrrole
DMAB: 4-dimethylaminobenzaldehyde
GABA: γ-aminobutyric acid
